# SURGNET: An Integrated Surgical Data Transmission System for Telesurgery

**DOI:** 10.1155/2009/435849

**Published:** 2009-05-26

**Authors:** Sriram Natarajan, Aura Ganz

**Affiliations:** Multimedia Networks Laboratory, Electrical and Computer Science Engineering, University of Massachusetts, Amherst, MA 01003, USA

## Abstract

Remote surgery information requires quick and reliable transmission between the surgeon and the patient site. However, the networks that interconnect the surgeon and patient sites are usually time varying and lossy which can cause packet loss and delay jitter. In this paper we propose SURGNET, a telesurgery system for which we developed the architecture, algorithms and implemented it on a testbed. The algorithms include adaptive packet prediction and buffer time adjustment techniques which reduce the negative effects caused by the lossy and time varying networks. To evaluate the proposed SURGNET system, at the therapist site, we implemented a therapist panel which controls the force feedback device movements and provides image analysis functionality. At the patient site we controlled a virtual reality applet built in Matlab. The varying network conditions were emulated using NISTNet emulator. Our results show that even for severe packet loss and variable delay jitter, the proposed integrated synchronization techniques significantly improve SURGNET performance.

## 1. Introduction

Remote Surgery allows the surgeon to perform surgery from remote locations without being physically present at the patient side. The high demands of this application include efficient surgical device robots and dedicated network links with reliable and high speed communication [1]. Surgeons in the US performed the first ever remote surgery, operating on a patient located in France via a trans-Atlantic link [2]. The surgery involves transferring of efficient medical information consisting of medical data such as real-time video, medical imaging, and operating surgical robots. When such real-time data with high reliability requirement is transmitted via lossy networks, the quality of temporal information gets degraded and affects the entire application.

In this paper we introduce SURGNET, a telesurgery system which enables efficient and reliable transfer of force feedback device information and medical images via lossy networks. Our system creates a closed loop architecture, that is, the update received from the remote patient side is used to perform the next movement of the force feedback device. The temporal information and irregular updates can cause the surgery device to move in the wrong direction restricting the purpose of the telesurgery. We propose algorithms that compensate for the network effects and ensure reliable transfer of such real-time data with two different synchronization techniques: intrastream and interstream. The intrastream synchronization technique modifies the output time or the rendering time at the receiver side to synchronize it with the source, and intermedia synchronization technique ensures that the closed loop structure of the architecture is maintained. In order to improve the intra-media synchronization, various research approaches have been proposed. In [3] the authors propose an integrated approach with a buffering scheme. In [4] the authors discuss the impact of delay jitter management and [5] discuss a moving average adaptive buffer. A dead reckoning technique which is introduced in [6] does not perform well in varying networking conditions. A predictor algorithm which is introduced in [7] linearly predicts the lost packets. However these works do not propose an integrated approach which improves the synchronization scheme for both packet loss and delay jitter. In this paper, we propose an integrated scheme which synchronizes force feedback data transmission over varying networking conditions dealing with both packet loss and delay jitter. 

On the therapist side, we run a Therapist Panel GUI which shows various features of the current position of the device to the surgeon. A detailed explanation of the panel is given in the server architecture presented in Section 2.1.1. At the patient side (described in Section 2.1.3) we have developed and implemented an enhanced adaptive predictor algorithm which employs an adaptive extrapolation and interpolation technique to predict the lost packets. Our scheme also compensates for the intrastream synchronization by modifying the playout time of the data packet. The algorithm involves enhanced time adjustment algorithm which improves the delay between consecutive packets. The varying network conditions are emulated using NISTNet emulator (see Section 2.1.2). 

The paper is organized as follows. In Section 2 we discuss SURGNET system which includes the functional description of the server (therapist) and client (patient) architectures as well as the NISTNet emulator. Section 3 describes the proposed synchronization techniques. Section 4 describes our experimental results, and Section 5 concludes the paper.

## 2. Remote Surgery Application

**Figure 1:**
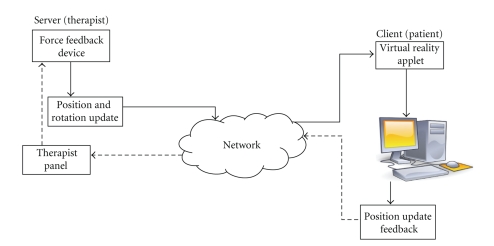
SURGNET architecture.

 SURGNET system architecture is depicted in Figure 1. The therapist operates the device from the server side which in turn moves the surgical device on the patient which is represented by an applet at the client side. The output of the applet can be used to update another device which performs the actual surgery on the patient. The updated positional information is sent at constant rate to the therapist wherein future movements of the device are controlled. The therapist can view the current position of the device on the Therapist Panel.

In the next subsection we provide a detailed description of the server and client architectures as well as the network emulator.

### 2.1. Server Side

#### 2.1.1. Server Architecture

**Figure 2:**
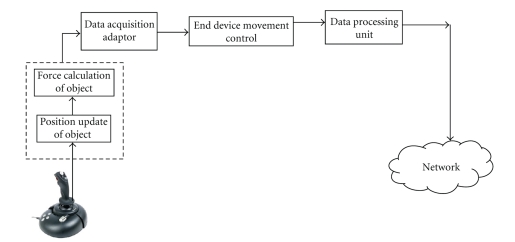
Server functional description.

Figure 2 describes the functional unit of the server. At the server side we have a force feedback device (Microsoft Sidewinder Joystick) that generates real-time data. The joystick is connected to the data acquisition adapter in Matlab. An ActiveX control communicates with the joystick reads the positional information of the device, and records the movement in both *X* and *Y* axes. The end device movement controls the maximum and minimum deviation of the device and transfers it to the data processing unit which packetizes the data as UDP and sends it to the network emulator. Since UDP is an unreliable transport protocol, we run Real-Time transport Protocol (RTP) over UDP to make the communication reliable.

**Figure 3:**
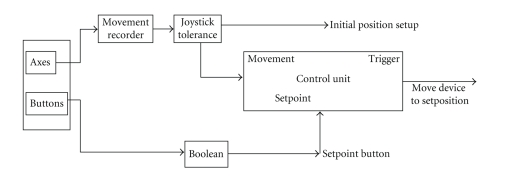
Data acquisition adapter.

A detailed description of the data acquisition adapter which is designed in Matlab is given in Figure 3. Here an ActiveX control interfaces between the device driver and the source of the adapter. The positional and force information is obtained from the device and is transformed to update the applet at the remote location. The data obtained from the device is transmitted as UDP packets with each packet containing the positional information of the device. We run an RTP over UDP to transfer the synchronization information which includes the sequence number of each packet and the timestamp of the occurrence of each positional data.

#### 2.1.2. Therapist Panel

**Figure 4:**
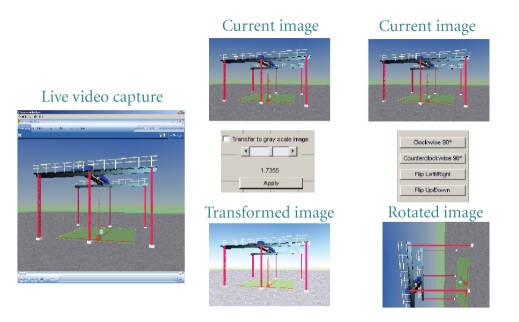
Snapshot of therapist panel GUI.

The therapist panel consists of image analysis techniques which enable the surgeon to perform various manipulations on the current image received from the client (patient) side. Such manipulations are required in order to identify the requirements of the next positional movement. Figure 4 shows a snapshot of the therapist panel which displays the current position of the device at the remote side. The panel operates in two different modes: Monitor mode and Playback mode. When the panel is operated in the Monitor mode, the updated data is captured at regular intervals from the remote side and is updated in the panel. When the surgeon decides to inspect and manipulate a particular image, the panel switches to the Playback mode, wherein the particular image is loaded for analysis. During this time the current data from the receiver side is received and stored in the buffer. Various image manipulations can be performed during the playback mode such as gray-scale transformation techniques which can control the brightness and contrast of the image. Region of Interest (ROI) is used to select one particular location of the image to consider for further processing. Image rotation is used to rotate the picture either in the clockwise or anticlockwise direction. The server will update the client (patient) side to modify the capture location and concentrate on a particular region for further analysis.

### 2.2. Network Emulator

The NISTNet network emulator [8] is a general-purpose tool for emulating performance dynamics in IP networks. The tool is designed to allow controlled reproducible experiments with network performance sensitive/adaptive applications and control protocols in a simple laboratory setting. By operating at the IP level, NISTNet can emulate the critical end-to-end performance characteristics imposed by various network situations. NISTNet Emulator intercepts the arriving packet and drops or introduces variable delay. Packets are modified based on the specification provided in the GUI supported by the emulator. In our experiments we tested SURGNET system with different packet loss percentages and delays. We used a Derivative Random Drop distribution which drops packets in a random fashion. The emulator which implements this functionality consists of an instrumented version of a live network implementation. NISTNet consists of two main parts: a loadable *kernel module*, which hooks into the normal Linux networking and real-time clock code, implements the run-time emulator and exports a set of control APIs; and a set of *user interfaces* which use the APIs to configure and control the operation of the kernel emulator. 

The NISTNet kernel module makes use of two hooks into the Linux kernel. In order to inspect all incoming packets for potential handling, the *packet intercept* code seizes control of the IP packet type handler. All IP packets received by network devices are then passed directly to the NISTNet module. After *packet matching* determines (based on the table of emulator entries) whether and how *packet processing* should affect the packet, NISTNet then (possibly after delay) passes the packet on to the Linux IP level code. The *fast timer* takes control of the system real-time clock and uses it as a timer source for *scheduling* delayed packets. In the process, it reprograms the clock to interrupt at a sufficiently high rate for fine-grained packet delays.

### 2.3. Client Side

#### 2.3.1. Client Architecture

**Figure 6:**
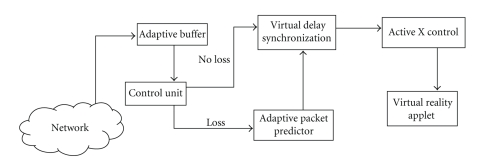
Client functional description.

The adaptive buffer at the client (patient) side receives the packet and passes it to the control unit which checks the arrival sequence of packets and compensates based on the loss or delay before updating the applet (see Figure 6). At the client side we have developed a Virtual Reality enabled browser applet. Updated position and force information from the joystick are used to control the movement in the applet. 

The delay variations and packet loss cause each data unit to arrive at the client with different time delays and varying packet loss. Due to the network impact, the data packets received at the client side do not match the data packets sent by the server, causing irregularities in the movement of the applet. To address these issues we propose an integrated synchronization scheme which is described in the next section.

#### 2.3.2. Control Unit Flow Chart

The Control Unit at the client side checks for the sequence of arriving packets to determine which synchronization algorithm to run. In case there is no loss but there is delay jitter we invoke the enhanced time adjustment algorithm that maintains 1 millisecond time difference between consecutive packets. If the time difference between consecutive packets is more than 1 millisecond, then time contraction is selected and if the difference is less than 1 millisecond, then time expansion is selected. When a packet is lost, the control unit runs the adaptive predictor algorithm to predict the missing positional value. The predicted value is passed to the buffer for further processing and applet update.

#### 2.3.3. Virtual Reality Applet

**Figure 8:**
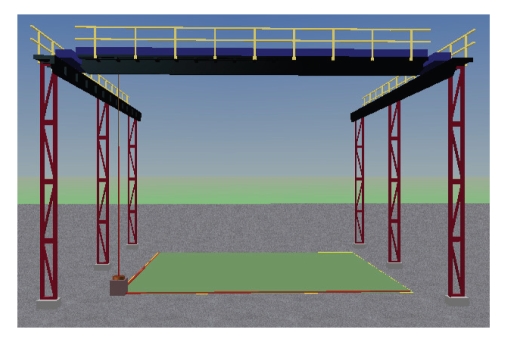
Applet at the client side.

Force feedback rendering is tested based on the synchronized movement of the two devices at the server and client sides. In our architecture we have implemented a Virtual Reality applet which runs on the browser on the client side (see Figure 8). The applet is similar to the vrcrane_joystick applet which is supported by the Matlab Virtual Reality toolbox. We have modified the applet to work in our application and enable the control from the remote application. When the source sidewinder joystick device is moved at the therapist side, we control the applet movement running on the client machine.

The applet which is developed in Matlab [9] contains a load hanging on the crane. The crane can move in *X* and *Z* axes. Here the *Y* axis movement is restricted and hence is not taken into consideration. Initial movement of the joystick will set the place where the load will move and when the force is applied then the actual movement of the load will take place. This will result in both the position and force value transferred from the server to the client side. In our application we have modified the vrcrane_joystick applet in Matlab by integrating force updates.

The joystick at the therapist side generates the data and sends it to the data acquisition adapter. The data is then processed and segregated into packets. Each packet contains both force as well as the position values. These UDP packets are sent through the emulated network. Once the data reaches the client side, the adaptive buffer will store it and process it in the control unit. The control unit decides whether to run the adaptive predictor algorithm, if there is a packet loss, or passes the packet to the Virtual Time Rendering Algorithm which determines when to update the applet based on the timestamp and adjusts the difference in the actual received time of consecutive data.

## 3. Synchronization Techniques 

Our compensation techniques involve a combination of an adaptive predictor algorithm and an enhanced time adjustment algorithm. The proposed synchronization schemes are discussed in detail in the next subsections.

### 3.1. Adaptive Predictor Algorithm

The force feedback data is time sensitive and used in real-time applications. Therefore, retransmission of lost packets is not desirable. In order to compensate for the packet loss, we implement an adaptive predictor algorithm which will employ extrapolation or interpolation techniques. When sufficient future packets are received we use interpolation techniques to predict the missing packet. In case a number of consecutive packets are lost we invoke extrapolation techniques. 

Packets are transmitted using the UDP protocol. Since UDP is unreliable, we run RTP over UDP to make a reliable connection. Since the packets are generated every millisecond, the receiver checks the sequence number of all arriving packets. Once it detects loss, the control unit sends the packet to the Predictor Unit which predicts the value of the position of the device based on the previously arrived packet. For Interpolation, a future packet is also considered. As the rate of change in position is lower than the rate at which the packets are generated, we determine the position of the lost packet based on the neighbor packets.

**Extrapolation Technique**

we denote packet *n* as *P*_*n*_ and assume that if that packet is lost, then the predictor determines the value of the lost packet based on the value of packets *P*_*n*−1_, *P*_*n*−2_. In our experiment, our results show better performance when considering the last two packets during extrapolation. The extrapolation prediction model is given by

(1) $$P_{n}=\sqrt{\frac{1}{n}\overset{n}{\underset{i=1}{\sum }}{\left(P_{n-i}\right)}^{2}},$$

where *n* = 2 (previous two packets are included).

The prediction model is a mathematical operation in estimating future values as a linear function. In (1) the root mean square criterion or the quadratic mean is calculated, which gives a statistical measure of the value of *P*_*n*_. This measure is specifically used when the variants are both positive and negative. In our experiment the *X*-axis position varies in both positive and negative direction and hence this technique predicts efficiently the closest value.

**Interpolation Technique**

If the buffer contains packet *P*_*n*+1_, we use this information to consider an interpolation technique to predict the lost packet. The interpolation prediction model is given by

(2) $$P_{n}=\sqrt{\frac{1}{n}\left[\overset{n}{\underset{i=1}{\sum }}{\left(P_{n-i}\right)}^{2}+{\left(P_{n+1}\right)}^{2}\right]},$$

where *n* = 3 (previous two packets and one future packet are included).

Equation (2) shows the calculation for interpolation where the quadratic mean uses three values. The optimization of the parameter can be adaptively selected and set to reduce the error. The data that is received in the buffer arrive at high data rate (1 millisecond) and hence considering the closest arrived packets in such calculations gives better results.

### 3.2. Enhanced Time Adjustment Algorithm

The Time Adjustment algorithm modifies the play out time of the packet which modifies the update rate of the applet to ensure that it synchronizes with the source. The intrapacket arrival time to the client side is variable. As explained above, in order to deliver to the applet the force feedback information as generated by the Joystick we need to keep a constant interpacket arrival delay of 1 millisecond. The proposed algorithm will adjust this time gap to 1 millisecond.

There are two cases that we have to consider: the time gap is smaller or larger than 1 millisecond. In such cases we need to run time expansion and time contraction, respectively. The time adjustment algorithm works similar to [9], but we do not assume any random process to the arrival of the data, as our approach runs the predictor algorithm. Adaptively the algorithm checks the arrival time of the packets and selects either virtual time contraction or virtual time expansion to adjust the intrapacket difference between the packets to be 1 millisecond. Before we describe the time adjustment algorithm, we first define the following terms:

- *ts*_*n*_—the timestamp of *n*th data when generated at the server,
- *tr*_*n*_—the timestamp of *n*th data when received at the client,
- *td*_*n*_—the timestamp of *n*th data when updated in the applet,
- *τ*—Packet processing delay at the receiver side.

The following inter-arrival times between two consecutive packets are defined as follows

- Source (at the server) inter-arrival time
  (3) $$Tsi=ts_{n+1}-ts_{n}.$$
- Received (at the client) inter-arrival time
  (4) $$Tri=tr_{n+1}-tr_{n}.$$
- Processed inter-arrival time
  (5) $$Td_{i}=td_{n+1}-td_{n}=\left(tr_{n+1}-tr_{n}+\tau \right).$$
- Time adjustment factor
  (6) $$\beta =Td_{i}-1.$$

The applet update time *Ta*_*i*+1_ uses the following time adjustment algorithm:

(7) $$Ta_{i+1\;}=td_{n+1}-\beta ;\;\text{if }\beta >0,$$

(8) $$Ta_{i+1}=td_{n+1}+\beta ;\;\text{if}\;\;\beta <0.$$

The buffering scheme receives the packets from the network, and the control unit checks for continuity of packets by analyzing the sequence number with the RTP information. If the packets arrive in order then the packets are passed to the time adjustment algorithm. Equation (4) calculates the time difference between the received sequential packets from the network. Each packet in the receiver takes different processing time, and (5) calculates the time change after processing. The applet update time is the time when the actual applet movement takes place corresponding to the movement of the force feedback device at the server side. Hence a time adjustment factor is required to readjust the time difference between the packets before updating the applet. By default the time difference between the packets should be maintained as 1 millisecond. The processed time depends on the time adjustment factor *β* that selects to run either the Time Contraction or Time Expansion to adaptively run and synchronize the server and client. The algorithm checks the timestamp value of the processed packets. If the time difference after processing time between the packets is more than 1 millisecond (*β* > 0), (7) Time Contraction is processed with the first packet time as reference time and adjusting the sequential packet time to 1 millisecond more. If the time difference is less than 1 millisecond (*β* < 0), (8) Time Expansion is processed with the first packet time as reference and adjusting the sequential packet time.

## 4. Experiments

### 4.1. Testbed

**Figure 9:**
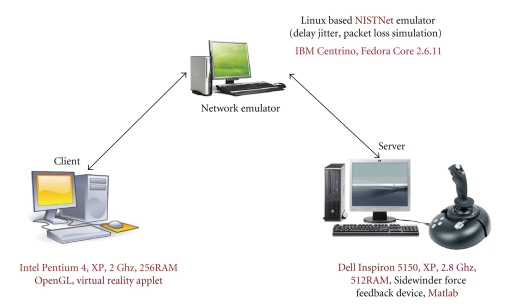
SURGNET testbed.

As depicted in Figure 9 the testbed consists of a Server (see Section 2.1), NISTNet Emulator (see Section 2.2) and a Client (see Section 2.3).

**Server**

The Microsoft Sidewinder Force Feedback Device which is connected to the Server has a rendering rate of 1 Khz. For convenience, we have only recorded *X*-axis movements for the joystick. The generated data *X*-axis position also moves in the negative axis due to the fact that the base of the handle in the Sidewinder Joystick is located in the center. We programmed the joystick to output negative values when the handle moves to the left and positive values when it moves to the right.

The Server runs the Matlab Processing Unit which takes input from the joystick and reads the Axis and button values from the data acquisition adapter. The server runs on a Dell Inspiron 5150 Windows XP machine, with 2.8 GHz and memory of 512 RAM.

**NISTNet Emulator**

The Network Emulator (NISTNet) is setup on an IBM Centrino, Linux machine. The machine is treated as a router which takes input from the server, processes the packets (drops and/or delays them), and sends them to the client side. NISTNet Emulator runs on a Fedora Core 2.6.11 machine with Real Time Clock setup as separate module from the Kernel.

**Client Side**

The Client side runs the Virtual Reality Applet. The Client side runs on an Intel Pentium 4, XP with 2 GHz and 256 of RAM Memory.

### 4.2. Experiments

**Case 1**

**Figure 10:**
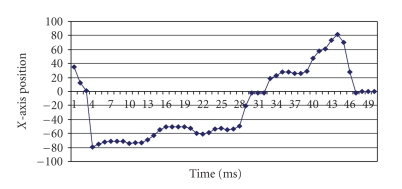
Data generated at server.

**Figure 11:**
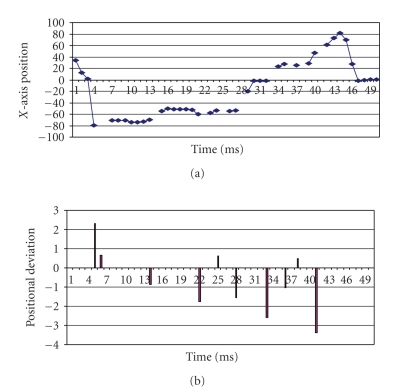
(a) Data received at client (20% loss) *unsynchronized.* (b) X-Axis positional deviation (20% Loss) *synchronized.*

**Figure 12:**
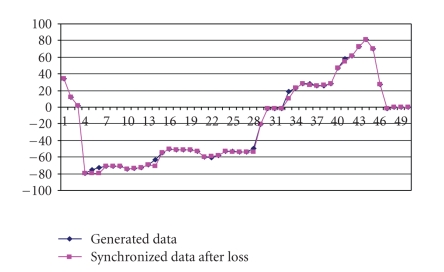
Comparison between generated data at server and *synchronized* data at client.

**Loss only.** We first verify the proposed synchronization algorithms for the case in which only loss occurs in the network. The server side data was generated by random movements of the joystick handle. Positional data variations are within the maximum limit of 100 in the positive axis and −100 in the negative axis. The data generated at the server side is shown in Figure 10. Figure 11(a) depicts the data received at the client side with 20% loss. The deviation between the generated data and the predicted data received at the client side is shown in Figure 11(b). Figure 12 which depicts the positional data comparison between the server and the client side shows an almost perfect synchronization (with the maximum percentage deviation of 2.89%.). We also calculated the maximum percentage deviation for various percentages of loss The results are tabulated in Table 1.

**Case 2**

**Figure 13:**
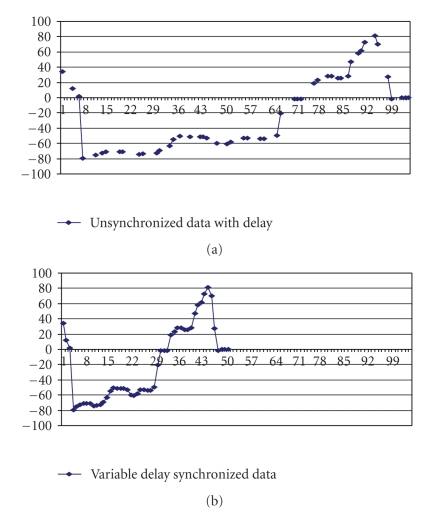
(a) Variable Delay *unsynchronized* data. (b) Variable Delay *synchronized* data.

**Delay jitter only.** The emulator introduces random variable delay for each packet sent from the server. For each packet sent from the server, the time difference between the packets should be 1 millisecond. Figure 13(a) shows the packets received at the client after the random delay has been introduced, and Figure 13(b) shows the time adjusted position which modifies the time difference between the packets to 1 millisecond.

**Case 3**

**Figure 14:**
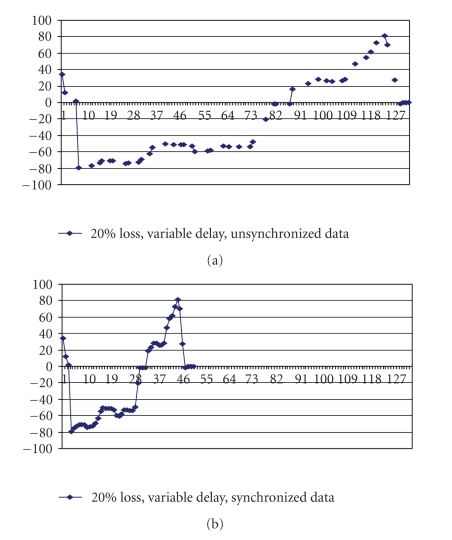
(a) 20% Loss with Variable delay, *unsynchronized* data. (b) 20% Loss with Variable delay, *synchronized* data.

**Figure 15:**
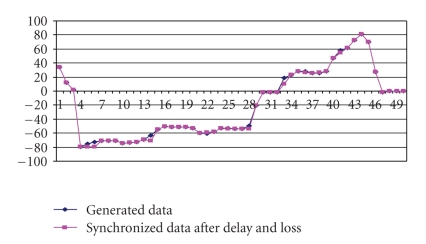
*X*-axis Positional Deviation Comparison at Server and Client Side.

**Loss and delay jitter.** Figure 14(a) shows both packet loss and delay jitter between packets. In our integrated approach the control unit predicts the lost packet and passes the packet to the Time Adjustment unit which modifies the playback time. Figure 14(b) shows the output of predicted packet with delay and the corresponding integrated synchronization scheme. Figure 15 gives a comparative analysis between the data generated at the server side and predicted and time adjusted data at the client side. The results indicate the efficiency of our integrated algorithms and show their effectiveness in controlling the applet by the force feedback device. The control unit first runs the packet predictor algorithm and then the time adjustment algorithm before updating the applet. The total delay for the first 50 packets was 127 milliseconds, but after readjusting the delay, the inter-packet delay between the packets is modified to 1 millisecond and hence the overlap occurs in time.

## 5. Conclusion and Future Work

We designed implemented, and tested SURGNET, an integrated remote surgery system. SURGNET enables the use of a force feedback device in remote surgery in spite of severe network impairments. The proposed algorithms address both the packet loss and delay jitter issues which are major constraints in time varying networks. We have also integrated medical image analysis functionality along with the force feedback data by introducing the Therapist panel. Our results show that in spite of severe loss and network delay conditions, the proposed algorithms successfully synchronized the applet at the patient side with the device on the therapist side, enabling a smooth manipulation of the surgical instruments. In our future work we plan to implement multicasting and group synchronization of devices in time varying networks.

## Figures and Tables

**Figure 5:**
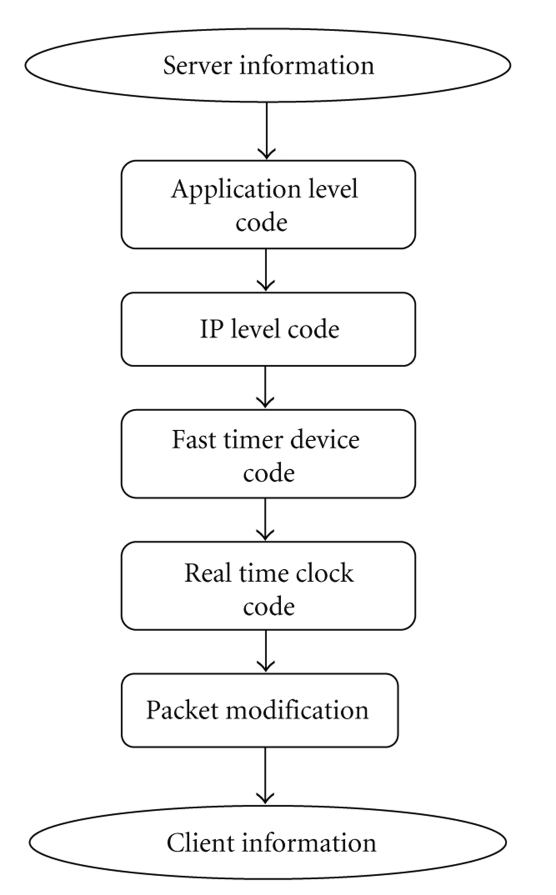
NISTNet emulator.

**Figure 7:**
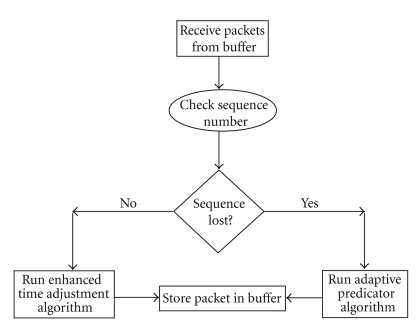
Control unit flow chart.

**Table 1:** Maximum percentage deviation of the predicted value of **X**-Axis position at client.

Loss	10%	15%	20%
Maximum Percentage Deviation	1.48%	2.21%	2.89%
